# Maximal Sum of Metabolic Exchange Fluxes Outperforms Biomass Yield as a Predictor of Growth Rate of Microorganisms

**DOI:** 10.1371/journal.pone.0098372

**Published:** 2014-05-27

**Authors:** Raphy Zarecki, Matthew A. Oberhardt, Keren Yizhak, Allon Wagner, Ella Shtifman Segal, Shiri Freilich, Christopher S. Henry, Uri Gophna, Eytan Ruppin

**Affiliations:** 1 School of Computer Sciences, and Sackler School of Medicine, Tel Aviv University, Tel Aviv, Israel; 2 Department of Molecular Microbiology and Biotechnology, Faculty of Life Sciences, Tel Aviv University, Tel Aviv, Israel; 3 Newe Ya'ar Research Center, Agricultural Research Organization, Ramat Yishay, Israel; 4 Mathematics and Computer Science Division, Argonne National Laboratory, Argonne, Illinois, United States of America; Virginia Commonwealth University, United States of America

## Abstract

Growth rate has long been considered one of the most valuable phenotypes that can be measured in cells. Aside from being highly accessible and informative in laboratory cultures, maximal growth rate is often a prime determinant of cellular fitness, and predicting phenotypes that underlie fitness is key to both understanding and manipulating life. Despite this, current methods for predicting microbial fitness typically focus on *yields* [e.g., predictions of biomass yield using **GE**nome-scale metabolic **M**odels (**GEMs**)] or notably require many empirical kinetic constants or substrate uptake rates, which render these methods ineffective in cases where fitness derives most directly from growth *rate*. Here we present a new method for predicting cellular growth rate, termed SUMEX, which does not require any empirical variables apart from a metabolic network (i.e., a GEM) and the growth medium. SUMEX is calculated by maximizing the **SUM** of molar **EX**change fluxes (hence SUMEX) in a genome-scale metabolic model. SUMEX successfully predicts relative microbial growth rates across species, environments, and genetic conditions, outperforming traditional cellular objectives (most notably, the convention assuming biomass maximization). The success of SUMEX suggests that the ability of a cell to catabolize substrates and produce a strong proton gradient enables fast cell growth. Easily applicable heuristics for predicting growth rate, such as what we demonstrate with SUMEX, may contribute to numerous medical and biotechnological goals, ranging from the engineering of faster-growing industrial strains, modeling of mixed ecological communities, and the inhibition of cancer growth.

## Background

In the data-rich landscape of present-day biology, large-scale network-based models are being increasingly tapped to make sense of the deluge of available data. Towards this end, genome-scale metabolic models (GEMs) have proven highly successful [1]. Incorporating gene-protein-reaction associations and stoichiometric reaction detail for the majority of known metabolic genes in an organism, GEMs have achieved high accuracies in predicting essentiality of gene knockouts (∼90%), growth phenotypes on a variety of substrates (∼90%) [2], and growth yields, and are useful tools in predicting metabolic fluxes [3]. These predictions typically rely on an assumption that single-celled organisms are optimized to maximize yield (for example: dry weight of biomass per unit of glucose consumed), following deep-rooted theories about evolutionary tuning towards optimal fitness [4]. However, as has been shown previously, maximization of molar yield is by no means a universal principle [5].

Metabolic phenotypes in GEMs are typically computed by a linear optimization method termed Flux Balance Analysis (FBA), in which a biomass objective is optimized while various network-defined constraints are upheld. Non-biomass objectives have also been tried, with varying powers of prediction [6], [7], [8], [9], but these objective functions are common in that they link metabolic models to growth yield or to a global flux distribution, rather than predicting growth *rate*. Recently, several works have incorporated large-scale data along with metabolic reconstructions into multi-system models, which include processes such as transcription, translation, growth rate dependence of the production of biomass constituents, and often vast numbers of empirical or approximated parameters [10], [11], [12], [13]. While some of these models are able to predict cell growth rates, they are all commonly limited in their reliance on a large amount of empirical data, as well as their reliance on high quality integrated models that would only be possible to produce for a select few extremely well studied organisms. Most notably, each of these models includes features that are varied (or tuned) in direct proportion to growth rate based on empirically measured quantities. The inclusion of these features means that these models leverage empirically provided *rate* data, which makes them impractical for predicting growth rates in circumstances in which such rate information cannot be systematically measured. This is inevitable, as without some rate information being added, GEMs are typically suited for predicting not growth *rates* but growth *yields*.

Growth *yield* (units of [g biomass produced]/[g substrate consumed]) is different from growth *rate* (units of 1/[hour]), although they are related by the substrate uptake rates of an organism growing at steady state (for growth on a single carbon source, for example, Growth rate  =  Substrate uptake rate * Yield). Prediction of yield using GEMs applies most rigorously to highly defined conditions such as in a chemostat in which one nutrient is limiting, and it is unclear how broadly applicable the ‘maximization of yield’ principle actually is [5]. In many conditions (including standard laboratory batch growth, growth of cancer cells displaying the Warburg effect, and competition of organisms for certain environmental niches), cells do not necessarily maximize their yield, yet their growth rate cannot be predicted without empirical data (e.g., substrate uptake rates). There is currently no framework for predicting cellular growth rates akin to the GEM-based methods available for predicting growth yields, which does not require extensive additional kinetic parameters. It would therefore be of significant value if a predictor of growth *rate* could be determined using genome-scale properties of GEMs that do not necessitate the arduous measurement of substrate uptake rates [14]. In a large number of conditions, especially in competitive niches, growth rate is a better measure for fitness than yield, so the ability to predict growth rates could significantly increase the utility of GEMs [15], [16], [17], [18].

## Results and Discussion

In this study we explore novel large-scale methods to predict variability in growth rates from GEMs grown on rich or defined media, and in some cases with gene knockouts. We focus on environments in which cells are expected to be optimizing their growth rate, such as maximal listed growth rates for species in rich media, or careful growth rate measurements of isogenic cultures in early exponential phase of batch growth. Our approach was inspired by an article by Vieira-Silva and Rocha [19], which investigated a number of bioinformatics-based measures for predicting the maximal growth rate across species. Vieira-Silva and Rocha collected from the literature the maximal growth rates in rich medium of over two hundred bacterial species, and then searched for a genomic measure that correlated best with these data. The genomic property of codon usage bias yielded their most promising correlation, but this property is not dependent on the growth medium, so it will fail when assessing growth rate of a species across media or other conditions. Furthermore, in cases of different cells of the same organism, such as human cancer cells, the cells share the same codons, and thus codon bias cannot be used to predict specific growth rate. It is possible that codon usage bias could be extended to predict growth rate under different conditions if, for example, it is recalculated only for the sets of genes highly expressed in a given medium. However, such work has not to our knowledge been done.

**Figure 1:**
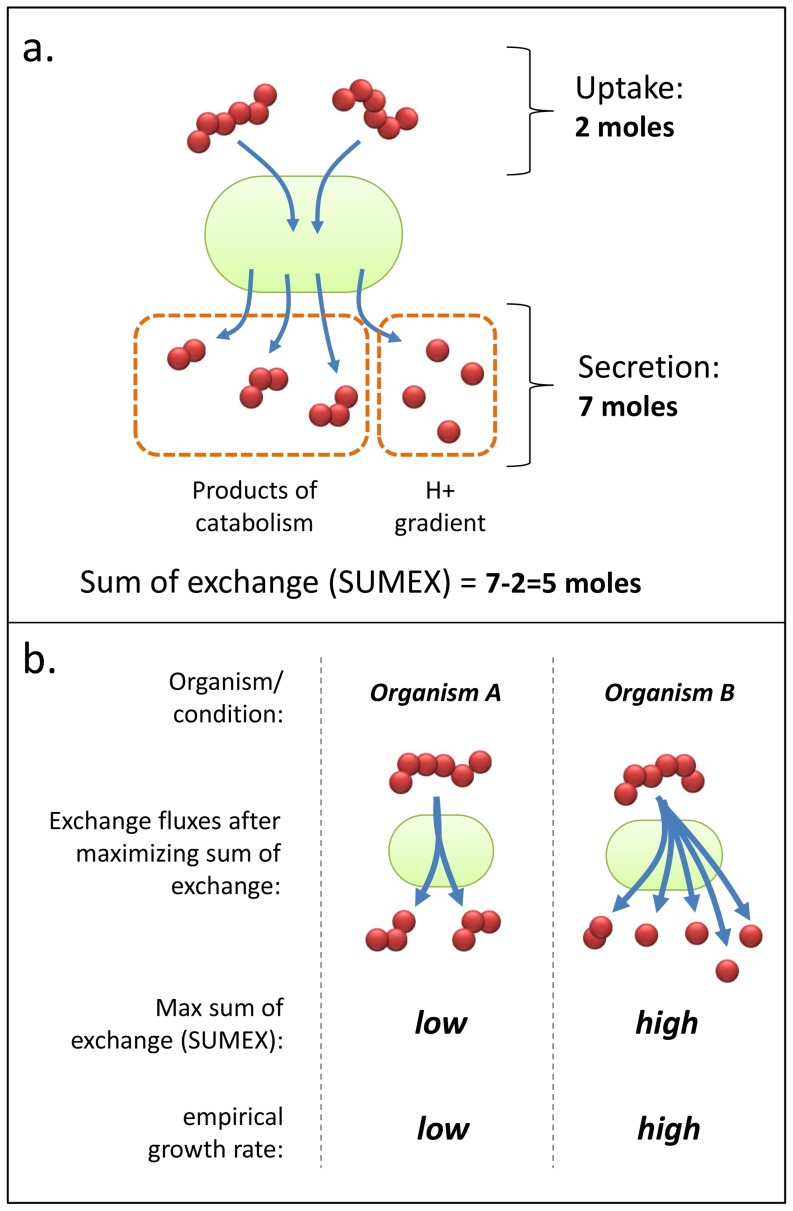
Schematic of SUMEX. (A) The summing of molar fluxes through exchange reactions, i.e., the quantity maximized in SUMEX, is illustrated. A high sum of exchanges value is achieved by high output fluxes and low input fluxes. Conceptually, this corresponds to a high catabolism, i.e., moles of substrates being broken down into multiple moles of smaller output molecules, and also a large production of extracellular protons. (B) SUMEX is the maximum possible sum of exchanges calculated in a particular organism and growth condition. Two theoretical organisms are compared here to display the observed correlation between a high SUMEX and a high empirical growth rate.

Analogously to *Vieira Silva* and *Rocha*, we explore a new class of metabolic objectives, related to maximizing the total metabolic secretion of a cell, which predict relative growth rate directly from GEMs. We focus on exchange fluxes because they are the missing gap between growth yield (which can be calculated relative to uptake rates by a GEM, e.g., in [2]) and growth rate (as stated before, Growth rate  =  Substrate uptake rate * Yield), and because there is an observed strong positive correlation between cellular surface-to-volume ratio and growth rate, as well as additional evidence suggesting that cell surface metabolism exerts most of the control of a cell over growth rate [20]. The exemplar of predictors we test is a novel method called “SUMEX,” which predicts growth rates of cells under different media conditions without requiring substrate uptake rates, kinetic constants, or any other empirical parameters. SUMEX is computed by maximizing the total molar output exchange minus input exchange of metabolites (which, given the sign convention in GEMs that all exchange reactions point outwards, is calculated as the ‘maximal **SUM** of **EX**change fluxes’), while setting a nominal lower bound on biomass production in order to ensure that some flux runs through biomass-producing pathways (see Fig. 1). A sensitivity analysis showed that the nominal bound on biomass (set at 5% of maximum) is not necessary for the performance of SUMEX in predicting growth rate, and that the predictiveness of SUMEX is insensitive to changes in this bound within a large range; nevertheless, we include it because it enforces biomass producing pathways must be able to carry flux under non-zero growth conditions (all of the datasets analyzed in this study are for non-lethal genetic conditions, so this condition is never relevant for comparisons of biomass vs. SUMEX done in this study; see Fig. S1 in File S1 and Fig. S2 in File S1).

SUMEX has a clear intuitive linkage to the concept of a cell's ability to do catabolism. A high SUMEX value indicates that a cell is capable of breaking down a small number of moles of its collective substrates (which include all compounds present in a medium that an organism is able to uptake) into a much larger number of moles of product (which include all excreted compounds), and a low SUMEX value indicates that the capacity of a cell to break down substrates in this manner is low (see Fig. 1a). Since SUMEX maximizes the sum of exchange fluxes of all exchangeable components available in the medium, it is important that the compounds in the growth medium are known. SUMEX is therefore a rough measure of the capacity of a cell to perform catabolism (see Fig. 1a), under a given medium condition. SUMEX is, in some sense, the simplest accounting of cell ‘catabolic capacity’ that does not take into account prior knowledge about the substrates or other features only available through condition-dependent experiments. It can thus be seen as a medium-dependent heuristic for catabolic capacity, which may shed potentially new light on the yield/growth rate relationship. In this sense, we do not necessarily expect that SUMEX will predict accurate intracellular fluxes, but rather consider it as a measure of a general network property of catabolism that may be predictive of growth rate. In this, SUMEX is similar to Biomass, which also is not generally very predictive of intracellular fluxes unless the model is subjected to auxiliary expression or flux constraints, or the condition is one for which the organism and model have been specifically adapted (e.g., *E. coli* in glucose minimal medium).

SUMEX represents a simple heuristic to maximizing catabolic activity of a cell, focusing exclusively on exchange reactions, and still ensuring a nominal production of biomass (we discuss a sensitivity analysis of this and other necessary bounds later in the paper, and in File S1).

The SUMEX formulation is: 
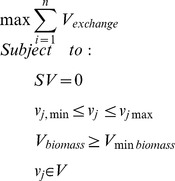



In which *v*'s correspond to metabolic flux values and *S* is the stoichiometric matrix, following GEM conventions. The formulation is explained in greater detail in the methods part of File S1.

**Figure 2:**
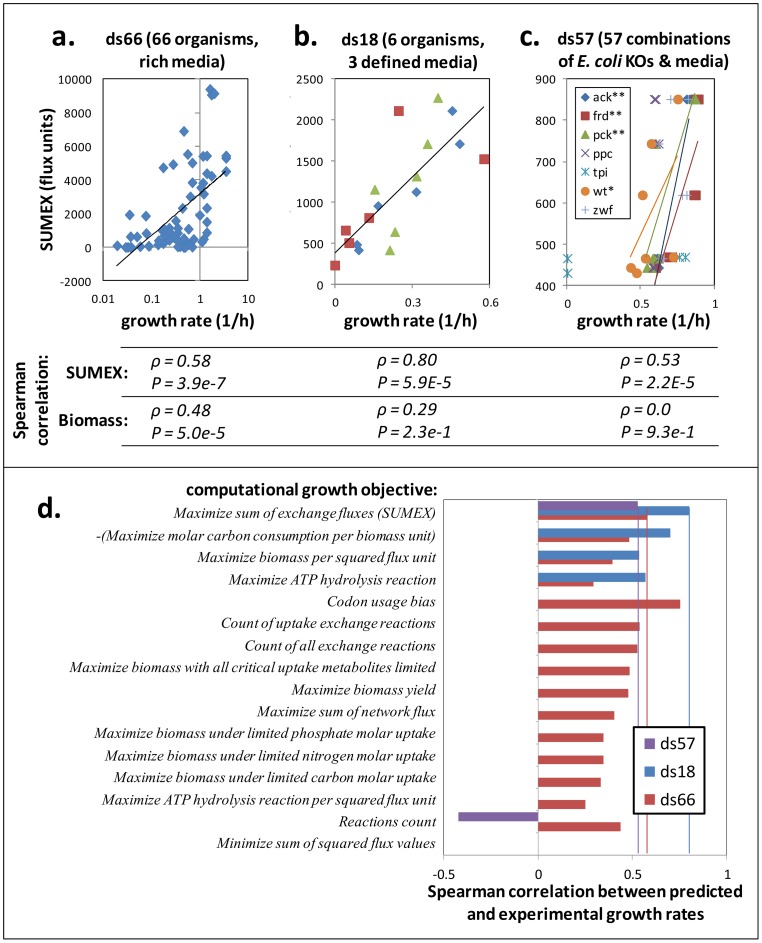
Correlation of different metrics to growth rate. (A–C) Spearman correlations of SUMEX vs. growth rate in three datasets. Colors in (B) represent media (green triangles, IMMxt; blue diamonds, IMM; red squares, IMM-gt; see Table S5 in File S1 for details). Colors in (C) represent strains. Trend-lines in (C) are shown for strains that individually show significance (*P≤5e-2, **P≤5e-3). Correlation values for SUMEX and Biomass vs. growth rate are listed below. (D) Significant (P-val ≤ 5e-2) Spearman correlations (i.e., ρ values) across three bacterial datasets for all tested metrics (non-significant correlations are not shown). Metrics are listed in descending order of the sum of ρ across the three datasets. Vertical lines denote rhos for SUMEX.

To test SUMEX and other methods, we collected two datasets of measured cellular growth rates from the literature: the previously mentioned *Vieira Silva* and *Rocha* dataset of maximal growth rates on rich media reported for 66 organisms (**ds66**) (see Table S2 in File S2) [19], and growth rates in early exponential phase of batch growth of 57 *Escherichia coli* wild type (WT) and knockout (KO) strains evolved for growth on a number of minimal media (**ds57**) [2]. We generated a third dataset in the lab, by measuring growth rates *in vitro* in the early exponential phase of batch cultures of 6 organisms on 3 defined media (**ds18**) (see Table S4 in File S1). Using automatically generated models from SEED [21], we then computed various growth-rate predictors for each of the models and conditions in these three datasets (ds66, ds57, and ds18). We compared SUMEX (as the exemplar of exchange-based metrics we had experimented with) against several metrics presented in a previous experimental study in *E. coli* of the optimal objectives of GEMs for predicting metabolic flux distributions [9]. Strikingly, SUMEX outperformed every previous metric in all three datasets in predicting variation in growth rates between different conditions, with only one exception in one dataset (codon usage bias from [19] correlated better than SUMEX with growth rates in ds66, but was non-predictive in the other datasets as it inherently cannot account for changes in the medium or gene knockouts). Most of the metrics correlated to some degree with growth rates on rich media (ds66), but all but three of them showed no significant correlation with growth rate in either of the defined datasets. Overall, SUMEX was the only metric among those tested to significantly correlate with growth rate across all three datasets, obtaining Spearman correlations of 0.58, 0.53, and 0.80 on ds66, ds57, and ds18 respectively, all with pvals < 6e-5 (see Fig. 2d; Pearson correlations were also significant, with rho  = 0.60, 0.40, and 0.78, and p = 8.3e-8, 2.0e-3, and 1.2e-4 for the 3 respective datasets).

Notably, the maximization of biomass yield, the aforementioned fitness metric used in hundreds of GEM studies, failed to significantly correlate with growth rates in two out of the three datasets (ds18 and ds57). This is despite previously noted strong correlations between GEM-predicted biomass yields and growth rates in ds57 when accounting for experimentally measured glucose uptake rates [2], which emphasizes the difference between predicting *rate* and predicting *yield*. In contrast, biomass yield was predictive of growth rate in ds66 (although not as predictive as SUMEX). This suggests that in rich media and when looking across a large range of organisms, both the growth rate and yield depend greatly on the capacity of an organism to take up many substrates – an observation supported by the relatively strong correlation between “count of uptake exchange reactions” and growth rate, as well as by the strong observed correlation between SUMEX and biomass yield, in ds66 (see Fig. 2a and Fig. S4 in File S1). Despite this, SUMEX correlates significantly with growth rate in ds66 even when controlling for biomass yield (ρ = 0.38, P = 1.6e-3 in partial Spearman correlation), showing that SUMEX provides information beyond that obtained from maximizing biomass. Biomass, on the other hand, does not correlate significantly with growth rate across ds66 when controlling for SUMEX (ρ = −0.18, P = 0.35). Surprisingly, maximization of ATP hydrolysis correlated poorly with growth rate, even though it has been previously shown to be predictive of intracellular fluxes in *E. coli* [9], [22]. These results suggest that while biomass and ATP hydrolysis are appropriate for measuring growth *yield*, they are not necessarily suited to measure growth rate using GEMs. A full description of metrics we tested is provided in File S1.

As previously mentioned, SUMEX requires no kinetic parameters, substrate uptake rates, or other empirical values to predict relative growth rates. To further benchmark SUMEX, we also tested it against previous methods for predicting growth rates that do require empirical parameters. A few such methods, which include several hundred kinetic constants or molecular crowding constraints, were introduced in recent years for *E. coli* [14], [23]. We tested the ability of SUMEX to predict growth rates reported in [14] for *E. coli* grown on 24 minimal media (henceforth: **ds24**), and achieved equivalent results to the state of the art (for consistency with the previous analyses, SUMEX was calculated for this dataset on the manually curated model, iAF1260 [24]; SUMEX and MOMENT, the method described in [14] and achieving the best previous result, each attained ρ = 0.47 and P = 0.02 in 2-sided Spearman tests; see Table S1 in File S1). Because SUMEX uses only the stoichiometry of metabolic reactions but no empirical parameters, it has the clear advantage that it can be easily computed across many species (if their metabolic models are available), as shown in the analyses of ds66 and ds18.

**Figure 3:**
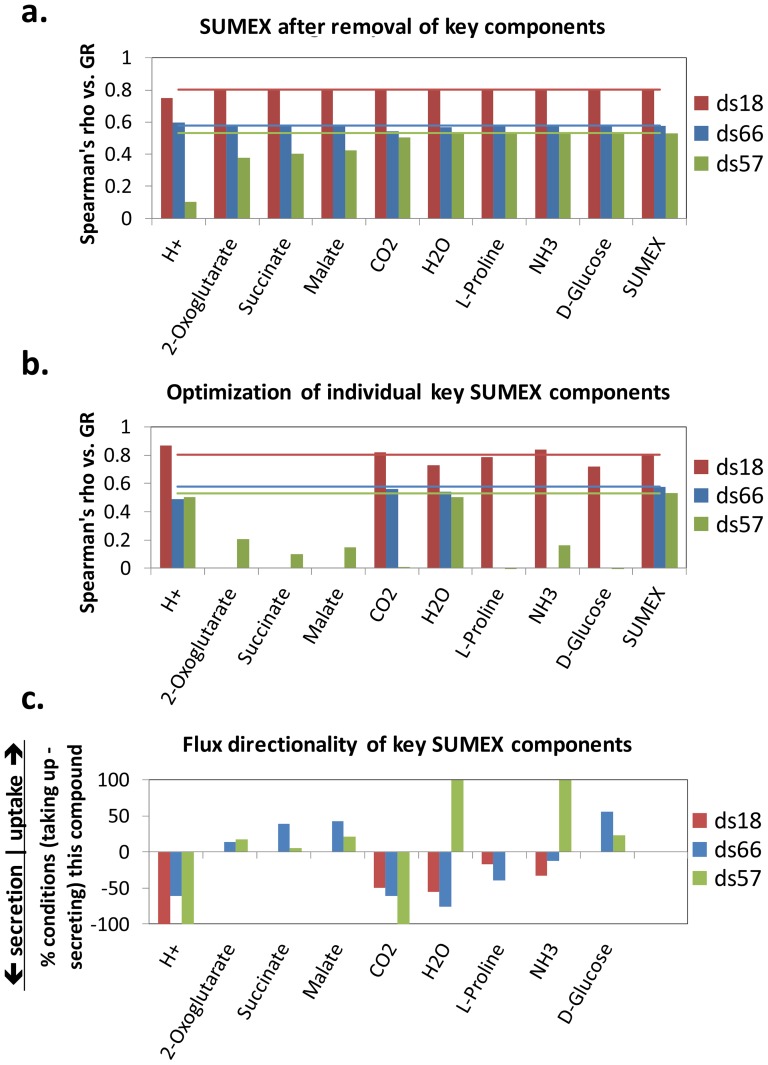
Component-wise analysis of SUMEX. (A–B) Spearman correlations of SUMEX versus growth rate (GR) across the 3 bacterial datasets when different exchange reactions are (A) removed from SUMEX or (B) optimized individually. Horizontal lines and rightmost set of columns show SUMEX ρ values. The components presented are all of those whose removal affected SUMEX ρ by >5% or that came within 5% of the SUMEX rho when maximized alone, for any of the 3 datasets. (C) The difference between the percent of models (per dataset) that must uptake vs. that must excrete a component in order to achieve maximal SUMEX.

To understand in more detail the mechanisms linking SUMEX to growth, we studied the relative contributions to SUMEX of different exchanged compounds. We did this by analyzing the effect of either leaving out or of individually optimizing the flux of each individual exchange metabolite. We found that the compounds that contribute most to SUMEX (those shown in Fig. 3) are H^+^ and several TCA-cycle intermediates, in addition to CO_2_. CO_2_, the main product of cellular catabolism, was necessarily released from the cell in nearly all conditions when SUMEX was optimized (Fig. 3C).

Interestingly, the removal of proton exchange from the SUMEX objective reduced the correlation of SUMEX with growth rate more than removal of any other component (it severely reduced the predictiveness of SUMEX in both ds18 and ds57 datasets – see Fig. 3A). Additionally, we found that maximizing the production of protons alone is nearly as predictive as SUMEX across the three bacterial datasets (see Fig 3B). Protons are the smallest metabolites in the metabolic models and can be readily produced from many different sources, and thus can account for a large portion of the total SUMEX flux (as we confirmed by flux variability analysis[25]). The strong correlation between maximal proton production and growth rate led us to hypothesize that if a cell has abundant resources for producing free extracellular protons, the strong resulting pH gradient may help drive ATP synthesis and gradient-driven transport, thus increasing overall growth rate and thus also contributing to the predictive power of SUMEX. It has been shown in *E. coli* and other species that when flux ranges are below saturation, the rate of ATP synthesis relates approximately linearly to the electrochemical gradient, which in respiring bacteria is determined primarily by the proton (i.e., pH) gradient [26], [27]. Therefore, we would expect the proton-related contribution to SUMEX to be more predictive in respirers than in obligate fermenters, for whom the production of ATP does not depend on the membrane gradient.

**Figure 4:**
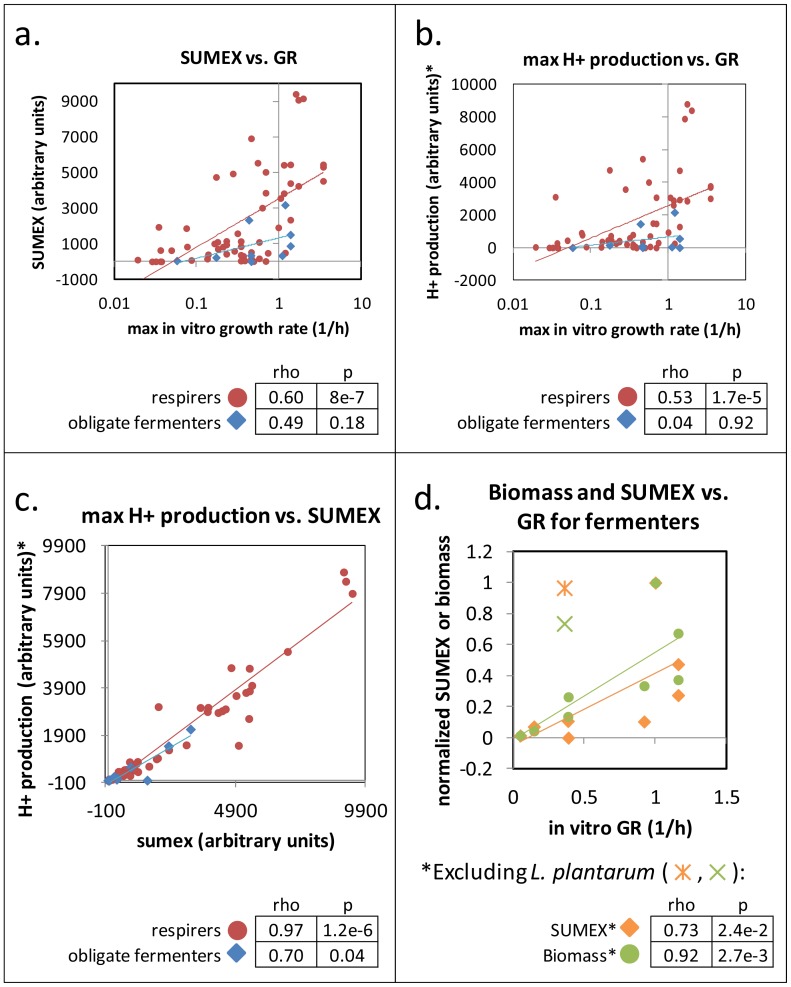
Prediction of growth in Respirers vs. Fermenters in ds66. Maximization of (A) SUMEX or (B) H+ production is plotted against growth rate for ds66 organisms, categorized into obligate fermenters (blue diamonds) and respirers (red circles) with trendlines shown. Rho and pvals are for 2-sided Spearman correlations. (C) Maximization of proton gradient correlates strongly with SUMEX in both respirers and fermenters. (D) SUMEX and Biomass as calculated on obligate fermenters are plotted vs. GR. Trendlines and Spearman correlations (1-sided) exclude L. plantarum, which can respire in the presence of heme and menaquinone (L. plantarum is shown on the plot as an orange asterisk (SUMEX) and a green “X” (Biomass)).

To test the fermenters vs. respirers hypothesis, we categorized the organisms in ds66 into two groups: 9 obligate fermenters (ds66f) and 57 organisms that can respire (ds66r). We found that the correlation of SUMEX with growth rate is stronger among only the respirers than among all organisms in ds66 (see Fig. 4a), that SUMEX is not significantly predictive of growth rate for obligate fermenters (also Fig. 4a), and that these same trends also apply when we instead compare maximization of proton production (PMAX) vs. growth rate (Fig. 4b). PMAX correlates strongly with SUMEX in models of both respiring and obligate fermenting organisms, despite the observation that neither is predictive of growth rate for obligate fermenters (see Fig 4c). This emphasizes the strong interdependency of SUMEX and PMAX.

When we remove a borderline case from the set of obligate fermenters (*Lactobacillus plantarum*, which has been shown to respire if provided heme and menaquinone [28]), both SUMEX and biomass maximization became predictive of fermenter growth rates (Fig. 4d). Therefore, a larger dataset of growth rates of obligate fermenters than currently at our disposal will be needed to unequivocally determine whether SUMEX can be used to predict relative growth rates of obligate fermenters. See our continued analysis in File S1.

**Figure 5:**
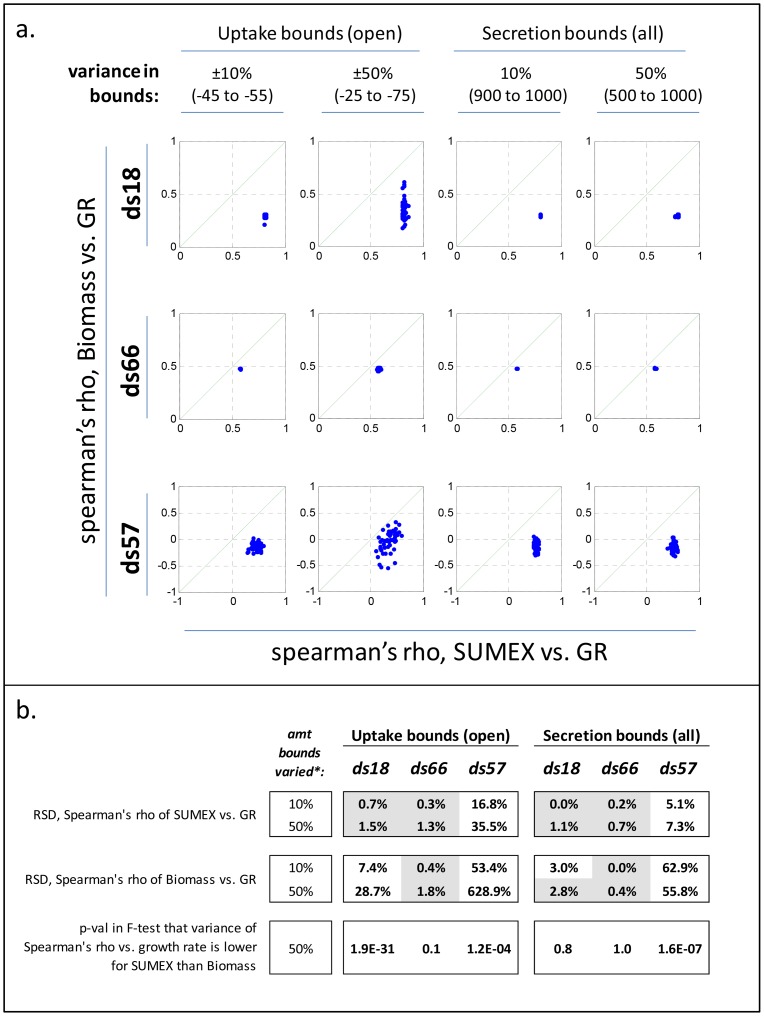
Sensitivity analysis of GEM bounds. (A) The Spearman's rhos (2-tailed) of growth rate versus both SUMEX (x-axis) and max Biomass (y-axis) are shown for 3 bacterial datasets (ds18, ds66, and ds57), when uptake bounds of all open metabolites (i.e., metabolites that are allowed to be taken up in a given medium) are randomly varied by ±10% (1^st^ column) or ±50% (2^nd^ column) of the standard bound (which is −50 for all allowed uptakes), and when secretion bounds of all exchanged metabolites are randomly varied by 10% (3^rd^ column) or 50% (4^th^ column) of the standard secretion bound (+1000). Sumex displays significant robustness to changes in bounds. The green line in each plot has a slope of 1. (B) Summary statistics from (A). The top four rows show the Relative Standard Deviation, RSD  =  abs((std(rho)/mean(rho)))*100, of SUMEX or Biomass versus GR across random variations in model uptake bounds or variations in secretion bounds (as labeled). Cases in which RSD is less than 10% of the variation in bounds are highlighted grey. The bottom row shows the significance (p-val) of an F-test that the correlation of SUMEX versus growth rate varies less across 50% variations in model bounds than the correlation of Biomass versus growth rate. The F-test shows high significance for uptake bounds in ds18 and ds57, and secretion bounds in ds57.

Limitations must be set on certain reaction bounds in a GEM in order to obtain feasible solutions (we used standard flux bounds of −50 for all allowed uptakes in SUMEX), which is a confounding factor in any attempt to produce parameter-less metrics in GEMs. Therefore, in order to ensure that the results seen for SUMEX are not simply due to the particular bounds we chose, we performed a sensitivity analysis. This test revealed that the correlation of SUMEX with growth rate is highly robust even up to 50% (or more) random variations imposed across all uptake (or secretion) bounds; we furthermore found that biomass is significantly less robust than SUMEX in 2 of the 3 datasets (see Fig. 5a–b).

The sensitivity of biomass to the bounds prompted us to try a follow-up analysis, in which we summed (but did not maximize) the mean allowed values of exchange fluxes given a constraint of optimal biomass. We did this only for models with randomly chosen uptake bounds that cause maximum biomass to correlate significantly with growth rate, which, in the absence of reliable full-organism exchange flux data, we assumed would be closer to the true exchange flux values than totally random bounds. This ‘sum of exchange’ (SUMofEX) variant on SUMEX also correlated significantly with growth rate in nearly every case, indicating that, if the exchange fluxes are known for an organism, merely summing them might be able to give an accurate value for growth rate (see Fig. S3 in File S1 and full description of SUMofEX in the Supplement).

## Conclusions

SUMEX represents a maximization of cellular catabolic activity as a cellular optimality principle, as outlined at the start of this paper. Notably, albeit its simplicity, SUMEX predictions correlate significantly with growth rate on every suitable dataset we were able to find in literature, as well as a set of growth rate data we measured ourselves. Our focus on developing a metric to predict relative growth rates given minimal empirical input stands in sharp contrast to recent trends in the GEM field, which have increasingly favored inclusion of large-scale datasets from multiple high-throughput sources and development of sophisticated models that are heavily tuned by empirical parameters (e.g., [10], [11], [12], [13]). This trend is justified for areas in which such data can be easily produced, but there is yet significant need for predictive, non empirically-tuned heuristics in areas where detailed measurements are infeasible or impractical. For example, SUMEX may be used to determine dynamics of microorganism-dominated ecosystems, in which most organisms may be very poorly understood, and their growth conditions may change over time. Building simple yet predictive models which require minimal empirical input is thus an important accompaniment to the data-driven models being developed. It will be interesting, additionally, to use data-driven models such as the ME model of *E. coli* [12] to analyze the proton gradient and the contribution of catabolic capacity, in order to better understand how exactly these capabilities contribute to growth rate.

SUMEX is clearly only a first step and a guiding concept for developing more predictive methods and insight, and likely forms only one piece of a larger emerging conceptual picture. A more sophisticated method utilizing the insights of SUMEX might incorporate the strengths of codon usage bias, SUMEX, and biomass yield maximization, and fit within a framework that incorporates all of them. We hope that this exploration of a promising alternate objective for predicting cell growth rate will stimulate future research in this area, and lead to better predictive models in the future.

## Materials and Methods

### Models

Unless otherwise noted, analyses were done on genome-scale metabolic reconstructions (GEMs) as obtained from SEED [21], at http://seed-viewer.theseed.org/. The 66 organisms in ds66 were chosen because (1) their GEMs were available from SEED and published in [21], and (2) their optimal doubling times were available from [19]. For analysis of ds24, the iJR904 *E. coli* model was used [29], and for analysis of E. coli expression, the *E. coli* SEED model was used. Table S2 in File S2 lists the names of the ds66 models and organisms. The non growth associated maintenance constraint was set to 0 for these analyses, in order to avoid artificially scaling model fluxes to a measured rate (since the non growth associated maintenance lower bound is usually set based on measured rate data from a chemostat).

### Implementation of growth rate predictors

Optimizations were run in *in silico* environments consistent with the known media, in which all exchange metabolites for a given species were available at a fixed rate of −50.0 (with output bounds of 1000). A sensitivity analysis was done to determine if these bounds affected the performance of SUMEX, and SUMEX was found to be robust to random changes in the bounds (and significantly more robust than biomass yield optimization; see Fig. 5a–b). In the case of ds66, the environment was ‘rich’, so we allowed uptake flux in all exchange reactions present in each organisms.

By convention, exchange fluxes denoting entrance of a metabolite into the cell (uptake) are negative valued, while exchanges denoting exit of a metabolite from the cell (output/secretion) are positive valued. Therefore, maximizing the total exchange flux (i.e. the SUMEX metric) would denote maximizing the output at the expense of the input (output exchanges – input exchanges). A full mathematical description of SUMEX is provided in File S1. The inclusion of a nonzero constraint on biomass yield (set at 5% unless otherwise mentioned) ensures that some flux is able to pass through biomass-required pathways; in cases where this constraint could not be met (i.e., because of the knockout of a lethal gene), the value of SUMEX was considered to be zero.

For simulation of maximal proton production (PMAX) (e.g., in Fig. 4), we increased the upper bound on proton production to +inf in order to avoid capping total protons produced. Manipulating this bound while running SUMEX did not significantly affect SUMEX results (data not shown). More details of the model constraints are provided in File S1.

### Technical implementation

Optimizations were done using the cplex tomlab optimization tool in a matlab environment. The free Gnu solver (GLPK) was checked in a few cases and returned identical results to the cplex solver, and could thus be easily exchanged. Most of the analyses were run using a standard desktop computer; a few were run in a computing cluster or on a high-powered linux machine. Sample Matlab code to run SUMEX is provided in Materials S1.

### Growth experiments of 6 organisms on 3 defined IMM media (ds18)

To validate SUMEX, we performed *in vitro* experiments to measure the growth rates of a number of organisms (listed in Table S3 in File S1) in multiple environments. Growth experiments were conducted in 96-well plates at 30°C, with continuous shaking, using a Biotek ELX808IU-PC microplate reader, on variants of IMM medium, as detailed in Tables S3 and S4. Optical density was measured every 15 minutes at a wavelength of 595 nm. Growth rates were determined during early to mid exponential growth phase by taking the slope of a linear fit through the natural log of the data.

## Supporting Information

File S1 Supplementary information, contains supplementary results and methods.
(DOCX)

File S2 Supplementary raw data files, and Table S2 (containing details on organisms in ds66).
(XLSX)

Materials S1 Zipped file containing matlab code to run SUMEX (read README.txt after unzipping).
(ZIP)
