# Supplementary material for: Maximal Sum of Metabolic Exchange Fluxes Outperforms Biomass Yield as a Predictor of Growth Rate of Microorganisms
Source: PLoS One. 2014 May 27;9(5):e98372. doi: 10.1371/journal.pone.0098372 (PMC4035307; doi:10.1371/journal.pone.0098372)
Supplement: File S1 — Supplementary information, contains supplementary results and methods. (DOCX) [file pone.0098372.s001.docx]

**Supplementary Materials:**

Contents

[I. Supplementary results 1](#_Toc376447676)

[A. Sensitivity analysis of SUMEX and Biomass 1](#_Toc376447677)

[B. Expanded analysis of obligate fermenters and respirers in ds66 2](#_Toc376447678)

[C. The relationship between flux and molecular weight in SUMEX: 3](#_Toc376447679)

[D. Ranging of biomass% lower bound: 4](#_Toc376447680)

[E. Network flexibility in SUMEX as biomass lower bound approaches 100%: 7](#_Toc376447681)

[F. Summing exchange fluxes in the optimal biomass solution space predicts growth rate: 9](#_Toc376447682)

[G. Gene Expression of pathways contributing to SUMEX: 11](#_Toc376447683)

[H. Correlation of SUMEX and Biomass: 14](#_Toc376447684)

[II. Supplementary Methods: 15](#_Toc376447685)

[A. Models: 15](#_Toc376447686)

[B. General methods: 15](#_Toc376447687)

[C. Reactions constraints and optimal environment setting: 16](#_Toc376447688)

[D. Building NCI60 cancer cell models: 16](#_Toc376447689)

[E. Computation of metrics: 17](#_Toc376447690)

[F. Growth experiments of 6 organisms on 3 defined IMM media (ds18): 25](#_Toc376447691)

[Table S3: Description of ds66 25](#_Toc376447692)

[Table S4: *in vitro* growth experiments (i.e., ds18) 25](#_Toc376447693)

[Table S5: IMM defined medium 27](#_Toc376447694)

# Supplementary results

### Sensitivity analysis of SUMEX and Biomass

SUMEX does not assume known uptake rates. This is an important strength of the metric, because only in cases where uptake rates of key compounds are known, can traditional methods (most notably, FBA using a biomass objective) predict growth rate due to the rate-yield relationship (*Growth rate = Substrate uptake rate * Yield*) (this is at least true in substrate limited conditions). However, in optimizing any objective function in a GEM (including SUMEX), it is necessary to set bounds on the uptake reactions (or at least on some reactions) in order to gain computationally feasible solutions. We chose to set standard bounds on uptakes of all compounds in a given medium at a value of -50 units (see Supplementary Methods for full characterization of the bounds). We set the same standard bounds for all metrics we tried, unless otherwise noted. In order to test how dependent SUMEX is on these bounds, we did a sensitivity analysis across the 3 datasets, testing both SUMEX and biomass. Briefly, we altered each uptake bound across all models in a given dataset by a random amount between either ±10% or ±50% (uniformly distributed) of its standard value, and then re-assessed the correlation of the metric against growth rates for that dataset (see Fig. 5).

We found the correlation of SUMEX with growth rate to be highly robust to changes in the uptake bounds, and indeed to be significantly more robust than biomass on two of the three datasets given the same random distributions of uptakes (P=2e-31 and P=2e-4 in F-tests on ds18 and ds57, respectively, at 50% variation; there was no distinguishable difference in ds66 – see Fig. 5b). In the rich media conditions of ds66, the correlation of SUMEX vs. GR varied less than 10% even with 100% variance in uptake bounds. For completeness, we repeated the same test on the secretion bounds and achieved similar results (see Fig. 5a-b).

### Expanded analysis of obligate fermenters and respirers in ds66

As noted in the main text, we split ds66 into two groups: obligate fermenters and organisms that respire (see Table S2 for the breakdown). We found that SUMEX is predictive of growth rate for the respirers, but not for the obligate fermenters. Of note, although SUMEX does not significantly correlate with growth rate for the 9 obligate fermenters, it also does not show significantly less significance than randomly chosen sets of 9 organisms from ds66. Unlike SUMEX, biomass yield does show a significant correlation versus GR for the 9 obligate fermenters (rho = 0.66, p = 0.03 in 1-sided Spearman test), although the significance is also not significantly above that expected if we choose 9 organisms at random. This suggests that while biomass is a poor predictor of growth rate in respirers, it may be appropriate for predicting the GR of obligate fermenters. Among the set of 9 obligate fermenters, there was one organism, *Lactobacillus plantarum*, for which evidence has been found for respiration when the organism is provided exogenous heme and menaquinone [[1](#_ENREF_1)]. Therefore, it is possible that *L. plantarum* should be re-categorized as a respirer. Removing *L. plantarum* from the fermenter set and calculating Spearman correlations on the remaining 8 organisms resulted in significance for both biomass and SUMEX versus GR (see Fig. 4d). Due to these considerations, a larger dataset of obligate fermenters will be required in order to allow more definite statements about the application of SUMEX or biomass to predicting their growth (none of the other datasets treated in this paper include obligate fermenters).

Interestingly, SUMEX and PMAX significantly under-predict the growth rates of obligate fermenters compared with respirers (all fermenter datapoints lie below the trendline in Fig 4a-b). This suggest that, since growth of fermenters relies on mechanisms independent from their ability to produce a strong proton gradient, a proton gradient-dependent predictor (such as SUMEX) under-represents their capability for fast growth.

### The relationship between flux and molecular weight in SUMEX:

In order to check if maximizing SUMEX indeed causes uptake of high molecular weight compounds and the output of low molecular weight compounds, we calculated the correlation between the molecular weights of exchanged metabolites (with nonzero fluxes) and their average exchange fluxes (as determined by flux variability analysis) when calculating SUMEX for E. coli on rich medium, as well as for all exchanged metabolites in all models across the ds18 dataset. We achieved strong negative correlations between molecular weight and outward exchange flux in both cases (ρ=-0.73, P = 4e-23 and ρ=-0.56, P = 1e-34 for the two analyses), confirming our hypothesis.

### Ranging of biomass% lower bound:

Cellular growth involves an intrinsic tradeoff between growth rate and biomass yield. In calculating SUMEX, we enforce a small flux (5% of the maximum possible in the given condition) through the biomass yield reaction, since some yield is necessary to sustain growth. In order to more fully understand the relationship of SUMEX with growth yield, we varied this lower bound on biomass yield between 0 and 100% of the maximum (i.e., the maximum biomass yield computed in the model on a given media) in all of the datasets dealt with in this paper.

In the bacterial datasets, we found the correlation of SUMEX with growth rate to be typically robust to changes in the yield, except for when biomass approaches 100%, at which point the correlation drops off in several datasets (see Fig. S1). This suggests that the correlation of SUMEX with growth rate is robust to changes in yields in the model, at least within physiological ranges [^16^](#_ENREF_16),[^24^](#_ENREF_24). On the contrary, the dropoff near 100% biomass yield poses an interesting parallel with the results of [[2](#_ENREF_2)] (see Fig. S1). Flux variability analysis of ds18 confirmed that flux variability in maximal SUMEX decreases as the lower bound on biomass yield increases from 70% to 100% (see Fig. S2).

Intriguingly, we found a peak in the correlation between SUMEX and growth rate in ds66, ds18 and ds24 (peaks were at 90%, 55%, and 80% max biomass for the three datasets; see Fig. S1). These peaks in correlation with growth rate suggest that certain percentages of maximum biomass yields may be dominant across the different conditions in each dataset.

|  |
| --- |
| **Fig. S1: Effect of biomass lower bound on SUMEX:** The correlation of SUMEX versus growth rate as lower bound (LB) on biomass is varied. (a) ds66, (b) ds18, (c) ds57, and (d) ds24 (*E. coli* grown on 24 carbon sources, from [[3](#_ENREF_3)]). ds24 was calculated with the iAF1260 *E. coli* model. |

### Network flexibility in SUMEX as biomass lower bound approaches 100%:

In order to assess the flexibility of the metabolic networks as biomass approaches 100%, we did a flux variability analysis (FVA) of all reactions in ds18 under optimal SUMEX conditions with biomass set to equal 100%, 90%, 80%, and 70% of its max, and then we assessed the change in flux variability (∆FV) of the flux range of each reaction across the 18 conditions (6 organisms x 3 media). The ∆FV metric was calculated for each reaction/condition as the slope (change) of the flux range when the biomass value increases from 70% to 100% of its max. A positive ∆FV means that, as biomass increases between 70% and 100%, the range within which fluxes of a given reaction can vary increase, and the magnitude of ∆FV indicate the strength of the increase/decrease. Fig. S2 shows the results: it is clear that FV decreases as biomass trends towards 100%.

|  |
| --- |
| **Fig. S2: Flux variability in SUMEX solution as function of biomass lower bound:** FVA was performed on the optimal solution space of SUMEX at lower bounds of biomass between 100% and 70%. Reactions whose flux variability increased or decreased more than a set cutoff are binned into pathways and plotted. Overall, this shows a general decrease in flux variability as biomass approaches 100%. |

|  |
| --- |
| **Table S1: Analysis of ds24.** We obtained growth rates of *E. coli* in batch culture under 24 minimal media conditions from [[3](#_ENREF_3)]. For this analysis, we used the iAF1260 *E. coli* model, in order to be consistent with the other metrics. SUMEX was computed over the 23 media for which the carbon source was present extracellularly in the standard iAF1260 model (the excluded metabolite was glucosamine). Values listed are for SUMEX with the standard 5% lower bound of biomass. Also listed is the maximization of extracellular proton production (PMAX), which displays significance, but below that of the top 3 metrics. |

### Summing exchange fluxes in the optimal biomass solution space predicts growth rate:

We were interested in doing an independent validation of SUMEX, based on changing uptake bounds in the model. Our logic is as follows:

In the bacterial datasets, we did not have detailed measurements of uptake and secretion fluxes. However, because of the property that biomass yield corresponds to growth rate at steady state *if* uptake rates are exactly known (from mass conservation: $\mu=-\frac{1}{m_{biomass}}\sum_{i} m_{i}v_{i}$, where *μ*=growth rate and *m_i_* and *v_i_* are the mass and flux of each exchanged component, *i*), we hypothesized that if we tune the uptake rates to increase the correlation of biomass with growth rate, we would bias towards realistic uptake rates and be able to independently validate SUMEX by summing, *but not maximizing*, exchange fluxes.

Restated, we summed extrapolated uptake and secretion rates *without doing a maximization*, rather than computing the maximum achievable sum (as in SUMEX). In practice, this meant a four step process:

1. sample *in silico* media variants (i.e., random variations on the uptake and secretion bounds).
2. Keep variants that give significant (P≤0.05) positive correlations between maximal biomass and growth rate. These are cases that we hypothesize are, on average, more likely than random to be similar to true experimental exchange fluxes.
3. In the case of these variants, set biomass to its optimal value. Then determine the mean of allowable flux range for all exchange fluxes, and sum these values across the entire cell model (SUMofEX). Note, this is not a maximization of the sum of exchange, but rather a summing of the exchange fluxes given the condition that biomass is set to its maximum value, and that it correlates with growth rate.
4. Check the correlation of SUMofEX vs. growth rate in these cases.

Because any individual flux vector supporting maximal biomass is not unique, we calculated SUMofEX by summing the means of the flux variability ranges (computed by FVA) of each exchange component, as calculated within the biomass solution space.

We did this for ds18, ds66, and ds57, and achieved significant correlations between SUMofEX (calculated within the maximal biomass yield solution space) and growth rate in all three datasets, and indeed generally stronger correlations than the biomass objective achieved on the same data, as shown by points being to the right of the green line in Fig. S3. This analysis again independently validated the correlation of the sum of exchange fluxes with growth rates.

|  |
| --- |
| **Fig. S3: Extrapolating bounds for biomass.** Allowed uptake bounds were randomly varied via uniform distribution in the range [-50, 0] across all models for (A) ds18, (B) ds66, and (C) ds57. The variation was done such that for a single iteration, the uptake lower bound of a compound C_1_ was fixed to the same randomly determined value for all conditions in which C_1_ could be taken up, while the uptake of a compound C_2_ would take a different randomly determined uptake than C_1_ across all models, etc. Maximum biomass yield was computed for each model. Then, the sum of exchange fluxes (SUMofEX) was computed as the sum of the means of the flux variability ranges (calculated by FVA) of all exchange reactions, under the condition of optimal biomass (i.e., SUMEX wasn’t maximized, but rather it was summed from the means of allowed exchange fluxes that support optimal Biomass). The plots show only media variants in which biomass correlated significantly (P≤0.05) to growth rate, as we conjectured that these points would give the most accurate uptake bounds. Each dot represents the correlation coefficient (Spearman ρ) of growth rate vs. SUMofEX (x-axis) or optimal biomass (y-axis) for a single variant of the medium uptake bounds. Dots show points for which SUMofEX correlates significantly with growth rate, and red crosses show points for which SUMofEX does not correlate significantly. The green lines have a slope of 1, so points to the right of the lines denote variants of the uptake bounds for which SUMofEX correlated better than biomass yield with growth rate. |

###

### Correlation of SUMEX and Biomass:

We calculated the correlation of SUMEX and Biomass (2-sided Spearman test), and found that they correlate highly significantly on ds66, weakly on ds57, and insignificantly on ds18 (see Fig. S4).

|  |
| --- |
| **Fig. S4: Correlation of Biomass with SUMEX.** Plots of SUMEX versus Biomass are presented for (a) ds66, (b) ds57, and (c) ds18, along with Spearman tests. |

# Supplementary Methods:

### Models:

Unless otherwise noted, analyses were done on genome-scale metabolic reconstructions (GEMs) as obtained from SEED [[5](#_ENREF_5)], at <http://seed-viewer.theseed.org/>. The 66 organisms in ds66 were chosen because (1) their GEMs were available from SEED and published in [[5](#_ENREF_5)], and (2) their optimal doubling times were available from [[6](#_ENREF_6)]. For analysis of ds24, the iAF1260 *E. coli* model was used. Table S2 lists the names of the ds66 models and organisms.

### General methods:

Linear Programming (LP) and Quadratic Programming (QP) calculations were done using IBM Cplex software on an Intel based machine running Linux. The Spearman correlation calculations and other analyses were done using either Matlab software or Java.

Optimizations were run in *in silico* environments consistent with the known media, where all exchange metabolites for a given species were available at a fixed rate of -50.0. In the case of ds66, the environment was ‘rich’, so we allowed uptake flux in all exchange reactions for all organisms. Other constraints are described in the following section.

By convention, exchange fluxes denoting entrance of a metabolite into the cell (uptake) are negative valued, while exchanges denoting exit of a metabolite from the cell (output / secretion) are positive valued. Therefore, maximizing the total exchange flux (i.e. the SUMEX metric) would denote maximizing the output at the expense of the input (output exchanges – input exchanges).

### Reactions constraints and optimal environment setting:

Unless stated differently we used the following constraints on the reactions fluxes, and in the definition of rich media:

For irreversible reactions:

Exchange reactions:

0 ≤ V_i,ex_ ≤ Vi, Max_ex (Vi, Max_ex = 1000)

Non exchange reactions:

0 ≤ V_i_ ≤ V_i, Max_ ( V_i, Max_ = 1000)

For reversible reactions:

Exchange reactions:

Vi, Min_ex ≤ V_i,ex_ ≤ Vi, Max_ex (Vi, Min_ex = -50 Vi, Max_ex = 1000)

Non exchange reactions:

V_i,Min_ ≤ V_i_ ≤ V_i, Max_ (V_i,Min_ = -1000 V_i,Max_ = 1000)

### Computation of metrics:

Following is an explanation of the exact way we calculated each of the metrics listed in Fig. 2D:

#### Sum of exchange fluxes (SUMEX):

The sum of exchange fluxes (SUMEX) follows this procedure:

1. In addition to standard uptake constraints (see previous sections), we set a lower limit on biomass yield at 5% of its maximum, as determined by FBA on the given medium.
2. We search within this space for the max achievable exchange flux (secretion – uptake; calculated as the sum of exchange fluxes). SUMEX is the optimal value.

This can be represented mathematically as:

Where S is the stoichiometric matrix of metabolites and V is the vector of reactions that together define the metabolic model. SV = 0 defines the steady state of the metabolic model, and the limits on V_i_ are as defined in the reactions constraints section. V_biomass_ is the flux through the biomass reaction, and V_maxbiomass_ is the maximal achievable biomass yield, as determined through maximization of the biomass objective function (see next section). Because all exchange fluxes by definition point outwards (i.e., positive flux denotes secretion), the sum of exchanges intrinsically minimizes metabolic uptake and maximizes metabolic secretion in a single optimization. In practice we exclude V_biomass_ from the V_exchange_ vector when calculating biomass, but adding V_biomass_ back in has no significant effect on the solution (since V_biomass_ is typically very small in the SUMEX solution). The process is illustrated schematically in Fig. S5.

#### Maximal biomass objective function

This is the standard method for determining maximal biomass yield in a given environment using GEMs. We have taken the biomass function defined by the automatic metabolic models generator [[5](#_ENREF_5)] and we calculated its value when each of the organisms was grown in its given media.

The objective function solved was:

Where S is the stoichiometric matrix of metabolites and V is the vector of reactions that together define the metabolic model. SV = 0 defines the steady state of the metabolic model, and the limits on V_i_ are as defined in the reactions constraints section. This metric has been described extensively elsewhere (e.g., [[7](#_ENREF_7),[8](#_ENREF_8)]).

#### Codon usage bias:

This metric was described in [[6](#_ENREF_6)]. Codon usage biases for the 66 organisms of interest for our study were kindly provided by Vieira-Silva and Rocha.

#### Uptake exchange reactions count

This topological metric provides a simple sum of the number of uptake exchange reactions in a model (i.e., exchange reactions through which flux can enter the organism).

#### All exchange reactions count

This topological metric provides a simple sum of the total number of exchange reactions of the organism.

#### Maximize biomass with all critical uptake metabolites limited

This metric assesses the maximal biomass achievable under a limited uptake environment. For this analysis, all critical uptake reactions had their fluxes limited to -10.0 (negative indicating entrance into the cell, by standard convention). ‘Critical’ uptake reactions are those whose metabolites are fully consumed when the organism is grown in an optimal environment. Other than the change in constraints, the maximization was identical to the maximization of biomass metric.

#### Minimize molar carbon consumption per biomass unit

This metric is predicated on the hypothesis that evolution has driven selection for the most efficient usage of carbon in production of biomass. It is based on a metric from [[9](#_ENREF_9)], except instead of ‘glucose’ we minimize molar carbon uptake, as our models are grown in complex media.

We calculated this objective function in 2 steps:

Step 1)

Calculate the maximum biomass of the organisms when grown in a given media (see biomass objective description for details).

Step 2)

Calculate the maximum of the sum of exchange reactions that contain carbon and that are able to carry flux while fixating the maximum biomass flux value.

**Note: Because of the sign conventions on fluxes, when maximizing the flux of uptake exchange reaction we are actually minimizing the uptake of the specific exchange metabolite represented by this reaction, as uptake fluxes have a negative value in our models.

The Linear program solved by the second step is:

Where V_sc_ is the group of uptake exchange reactions that are able to carry flux and that contain carbon in their exchange metabolite.

#### Reactions count

Here we took the total count of reactions in the model, with the idea that a larger metabolism might correlate with a faster growth rate.

#### Maximize sum of network flux

Here we determined the sum of fluxes in the network, as an indicator of the general activity level of the metabolic network. We computed as follows:

#### Maximal biomass per squared flux unit

This method assesses the ability of a GEM to produce biomass while minimizing enzyme usage, as measured through the following formula:

.

We calculated this objective function in 2 steps:

Step 1)

Maximum biomass was calculated in an optimal environment.

Step 2)

Fixing biomass to its maximum value, we minimized the squared sum of all fluxes of the organism:

#### Maximize biomass under limited phosphate molar uptake

For this metric and a number of others, we assessed maximal biomass under limited nutrient uptake conditions. In this metric, we limited phosphate uptake. Specifically, we solved the following optimization problem:

Where Phosphate_count (V_p_) is the molar count of phosphate in the uptake exchange reactions that are able to carry flux.

We limited the total molar amount of phosphate to a value of -10.0, as we observed that providing higher levels did not limit growth, while reducing the limit was too limiting for some organisms, leading to minimal growth and reduced correlation.

#### Maximize biomass under limited nitrogen molar uptake

This metric is the same as the phosphate limitation metric, except nitrogen is limited instead. We limited the total molar amount of nitrogen to a value of -100.0 for the rationale described for the phosphate limitation metric.

#### Maximize biomass under limited carbon molar uptake

This metric is the same as the phosphate limitation metric, except carbon is limited instead. We limited the total molar amount of carbon to a value of -1000.0 for the rationale described for the phosphate limitation metric.

#### Maximize ATP maintenance (i.e., hydrolysis) reaction

This metric assesses the maximal molar amount of ATP that can be charged from ADP in the cell, given a set of inputs as media. ATP production, which is a measure of efficiency of energy production, is often considered as an alternative metric to biomass in genome-scale models. Production of more energy from a fixed set of cellular uptakes would thus logically be associated with stronger or faster growth.

The rationale behind this metric is that evolution drives maximal energetic efficiency. As none of the models contained an ATP maintenance (i.e., hydrolysis) reaction we added that reaction:

ATP + H2O -> ADP + H + Phosphate.

The linear problem computed is to maximize this ATP hydrolysis (also called ‘ATP maintenance’) reaction:

(Where V_atp_ is the ATP maintenance reaction).

#### Maximal ATP maintenance (i.e., hydrolysis) per squared flux unit

This method is based on a hypothesis that cells operate to maximize ATP maintenance yield (ie, the total amount of ATP that can be charged in a given environment) while minimizing enzyme usage. The total metric can be stated as follows:

We calculated this objective function in 2 steps:

Step 1)

Calculate maximum ATP maintenance flux as described under ‘Maximize ATP maintenance reaction.’

Step 2)

Calculate the minimum square sum of all fluxes of the organism when we fixate the ATP maintenance of the organism, using the following optimization:

where V_all reaction_ is the set of all the reactions in the metabolic model of the organism.

### Growth experiments of 6 organisms on 3 defined IMM media (ds18):

To validate SUMEX, we performed *in vitro* experiments to measure the growth rates of a number of organisms (listed in Table S3) in multiple environments. Growth experiments were conducted in 96-well plates at 30°C, with continuous shaking, using a Biotek ELX808IU-PC microplate reader. Optical density was measured every 15 minutes at a wavelength of 595nm. Growth rates were determined during early to mid exponential growth phase by taking the slope of a linear fit through the natural log of the data.

Using models taken from SEED [[5](#_ENREF_5)], we calculated various growth metrics (see Fig. 2D) in *in silico* environments mirroring the environments from the *in vitro* experiments. Table S4 contains the environment [[10](#_ENREF_10)] used *in vitro* (and *in silico*) and the changes done to it in the different experiments.

# Table S2: Description of ds66

Description of the 66 organisms that were used in the article, including categorization into respirers and obligate fermenters (and the sources used to determine those categories). Biomass and doubling times are for growth in an optimal environment. (The doubling times are from [[6](#_ENREF_6)]). The table is provided in a separate excel document.

# Table S3: *in vitro* growth experiments (i.e., ds18)

This table provides a list of *in vitro* growth experiments performed in our lab for validation of SUMEX. The table lists the species and the environments used. Simulations for *Serratia marcescens* were done using an *in silico* model for *S. odorifera* 4Rx13.796.

# Table S4: IMM defined medium

This table provides the IMM defined medium [[10](#_ENREF_10)] and its *in silico* representation. IMM was also modified to generate two alternate media.

| *Metabolite* | *In vitro medium* | *In silico medium* |
| --- | --- | --- |
| Thiamin | + | + |
| D-Methionine | + | + |
| Magnesium | + | + |
| L-Valine | + | + |
| L-Isoleucine | + | + |
| L-Leucine | + | + |
| L-Histidine | + | + |
| Calcium | + | + |
| D-Glucose-6-phosphate | + | + |
| Potassium | + | + |
| Citrate | + | + |
| L-Arginine | + | + |
| L-Tryptophan | + | + |
| L-Phenylalanine | + | + |
| Biotin | + | + |
| Riboflavin | + | + |
| Adenine | + | + |
| Pyridoxal | + | + |
| Nicotinamide_D-ribonucleotide | + | + |
| L-Glutamine | + | + |
| L-Cysteine | + | + |
| Lipoic acid | + | - |
| para-aminobenzoic acid | + | - |
| Oxygen | + | + |
| Cytosine | - | + |
| Zinc | - | + |
| Cobalt | - | + |
| Fe2+ | - | + |
| Chloride | - | + |
| Sulfate | - | + |
| Copper2 | - | + |
| Manganese | - | + |
| Spermidine | - | + |
| gly-asn-L | - | + |
| sn-Glycerol-3-phosphate | - | + |
| Octadecanoate | - | + |
|  |  |  |

**Additions done for the enlarged IMM environment (IMMxt):**

Xylose C5H10O5

Deoxythymidine C10H14N2O5

**Removals done for the reduced IMM environment (IMM-gt):**

Thiamin C12H17N4OS

D-Glucose_6-phosphate C6H12O9P

1. Brooijmans R, Smit B, Santos F, van Riel J, de Vos WM, et al. (2009) Heme and menaquinone induced electron transport in lactic acid bacteria. Microb Cell Fact 8: 28.

2. Schuetz R, Zamboni N, Zampieri M, Heinemann M, Sauer U (2012) Multidimensional optimality of microbial metabolism. Science 336: 601-604.

3. Adadi R, Volkmer B, Milo R, Heinemann M, Shlomi T (2012) Prediction of Microbial Growth Rate versus Biomass Yield by a Metabolic Network with Kinetic Parameters. PLoS Comput Biol 8: e1002575.

4. Wagner A, Zarecki R, Reshef L, Gochev C, Sorek R, et al. (2013) Computational evaluation of cellular metabolic costs successfully predicts genes whose expression is deleterious. Proc Natl Acad Sci U S A 110: 19166-19171.

5. Henry CS, DeJongh M, Best AA, Frybarger PM, Linsay B, et al. (2010) High-throughput generation, optimization and analysis of genome-scale metabolic models. Nat Biotechnol 28: 977-982.

6. Vieira-Silva S, Rocha EP (2010) The systemic imprint of growth and its uses in ecological (meta)genomics. PLoS Genet 6: e1000808.

7. Oberhardt MA, Chavali AK, Papin JA (2009) Flux balance analysis: interrogating genome-scale metabolic networks. Methods Mol Biol 500: 61-80.

8. Orth JD, Thiele I, Palsson BO (2010) What is flux balance analysis? Nat Biotechnol 28: 245-248.

9. Schuetz R, Kuepfer L, Sauer U (2007) Systematic evaluation of objective functions for predicting intracellular fluxes in Escherichia coli. Mol Syst Biol 3: 119.

10. Phan-Thanh L, Gormon T (1997) A chemically defined minimal medium for the optimal culture of Listeria. Int J Food Microbiol 35: 91-95.
